# Atherogenic index of plasma and coronary artery calcification progression beyond traditional risk factors according to baseline coronary artery calcium score

**DOI:** 10.1038/s41598-020-78350-x

**Published:** 2020-12-07

**Authors:** Ki-Bum Won, Donghee Han, Ji Hyun Lee, Su-Yeon Choi, Eun Ju Chun, Sung Hak Park, Hae-Won Han, Jidong Sung, Hae Ok Jung, Hyuk-Jae Chang

**Affiliations:** 1 Division of Cardiology, Ulsan University Hospital, University of Ulsan College of Medicine, Ulsan, South Korea; 2 Division of Cardiology, Severance Cardiovascular Hospital, Yonsei-Cedars-Sinai Integrative Cardiovascular Imaging Research Center, Yonsei University College of Medicine, Yonsei University Health System, 50-1 Yonsei-ro, Seodaemun-gu, Seoul, 03722 South Korea; 3 Department of Imaging and Medicine, Cedars Sinai Medical Center, Los Angeles, CA USA; 4 Division of Cardiology, Myongji Hospital, Ilsan, South Korea; 5 Division of Cardiology, Healthcare System Gangnam Center, Seoul National University Hospital, Seoul, South Korea; 6 Division of Radiology, Seoul National University Bundang Hospital, Seongnam, South Korea; 7 Division of Radiology, Gangnam Heartscan Clinic, Seoul, South Korea; 8 Department of Internal Medicine, Gangnam Heartscan Clinic, Seoul, South Korea; 9 Division of Cardiology, Heart Stroke and Vascular Institute, Samsung Medical Center, Seoul, South Korea; 10 Division of Cardiology, Seoul St. Mary’s Hospital, College of Medicine, The Catholic University of Korea, Seoul, South Korea

**Keywords:** Biomarkers, Cardiology, Diseases, Health care, Risk factors

## Abstract

This study aimed to evaluate the association between the atherogenic index of plasma (AIP), which has been suggested as a novel marker for atherosclerosis, and coronary artery calcification (CAC) progression according to the baseline coronary artery calcium score (CACS). We included 12,326 asymptomatic Korean adults who underwent at least two CAC evaluations from December 2012 to August 2016. Participants were stratified into four groups according to AIP quartiles, which were determined by the log of (triglyceride/high-density lipoprotein cholesterol). Baseline CACSs were divided into three groups: 0, 1 − 100, and > 100. CAC progression was defined as a difference ≥ 2.5 between the square roots (√) of the baseline and follow-up CACSs (Δ√transformed CACS). Annualized Δ√transformed CACS was defined as Δ√transformed CACS divided by the inter-scan period. During a mean 3.3-year follow-up period, the overall incidence of CAC progression was 30.6%. The incidences of CAC progression and annualized Δ√transformed CACS were markedly elevated with increasing AIP quartile in participants with baseline CACSs of 0 and 1 − 100, but not in those with a baseline CACS > 100. The AIP level was associated with the annualized Δ√transformed CACS in participants with baseline CACSs of 0 (β = 0.016; *P* < 0.001) and 1 − 100 (β = 0.035; *P* < 0.001), but not in those with baseline CACS > 100 (β = 0.032; *P* = 0.385). After adjusting for traditional risk factors, the AIP was significantly associated with CAC progression in those with baseline CACS ≤ 100. The AIP has value for predicting CAC progression in asymptomatic adults without heavy baseline CAC.

## Introduction

Coronary artery calcification (CAC) is associated with atherosclerotic burden and adverse cardiovascular (CV) clinical outcomes^1‒3^. Furthermore, CAC progression is a powerful predictor of mortality beyond traditional CV risk factors^4^. Recently, the Heinz Nixdorf Recall (HNR) study suggested that CAC progression may only offer additional prognostic benefit in the asymptomatic adult population, and that what counts is the baseline coronary artery calcium score (CACS) and risk factor assessment^5^. In particular, additional CAC evaluation was not recommended in cases of heavy baseline CAC, although a high CV risk was present in the HNR study^5^. This result suggests that early detection of the presence and progression of subclinical atherosclerosis is important in conditions with low to intermediate CV risk burden.

The atherogenic lipoprotein profile of plasma is a substantial risk factor for atherosclerosis. The atherogenic index of plasma (AIP) has been considered a marker of plasma atherogenicity based on its strong and positive association with lipoprotein particle size, cholesterol esterification rates, and remnant lipoproteinemia^6–8^. Notably, evidence suggests that the AIP may be more closely related to CV risk than are other atherogenic indices or individual lipoprotein cholesterol concentrations alone^9–11^. However, there is a paucity of data on the association between the AIP and CAC progression according to the baseline CACS. Thus, the present study aimed to evaluate the association between the AIP and CAC progression, focusing on the baseline CACS.

## Methods

### Study population and design

Data from the Korea Initiatives on Coronary Artery Calcification (KOICA) registry were analyzed in this study. The KOICA registry is a retrospective, observational, and multicenter registry of self-referred asymptomatic adults who underwent a general health examination at six healthcare centers in South Korea^12,13^. Briefly, the present study included 12,326 participants who underwent at least two CAC scan examinations, with available data on the AIP and diabetic status, from December 2012 to August 2016. All data were obtained during visits to each healthcare center. Self-reported medical questionnaires were used to obtain information on the participant’s medical history. Blood pressure of the right arm was measured using an automatic manometer, after resting for at least 5 min. Weight and height were measured with the participants wearing light clothing without shoes. The body mass index (BMI) was calculated as the weight in kilograms divided by the square of the height in meters. All blood samples were obtained after at least 8 h of fasting and analyzed for total cholesterol, triglyceride, high-density lipoprotein cholesterol (HDL-C), low-density lipoprotein cholesterol (LDL-C), creatinine, glucose, and glycated hemoglobin A1C (HbA1C) levels. Hypertension was defined as systolic blood pressure (BP) ≥ 140 mmHg or diastolic BP ≥ 90 mmHg, a previous diagnosis of hypertension, or use of anti-hypertensive medication. Diabetes was defined as either a fasting glucose level ≥ 126 mg/dL, HbA1C level ≥ 6.5%, a referral diagnosis of diabetes, or use of anti-diabetic treatment. Hyperlipidemia was defined as a total cholesterol level ≥ 240 mg/dL or anti-hyperlipidemic treatment. Obesity was defined as BMI ≥ 25.0 kg/m^2^ using cut-offs for the Asian population. Current smoking history was considered present if participants currently smoked or smoked until 1 month before the study. Hypertension, diabetes, hyperlipidemia, obesity, and current smoking status were considered as traditional risk factors. The AIP was defined as the base 10 logarithm of the ratio of the concentration of triglycerides to HDL-C^6^. All participants were categorized into four-groups based on AIP quartiles.

CACS was measured using the scoring system described by Agatston et al.^14^. CAC progression was defined using the SQRT method; specifically, as a difference ≥ 2.5 between the square roots (√) of the baseline and follow-up CACSs (Δ√transformed CACS), with consideration of the inter-scan variability^4,15^. Annualized Δ√transformed CACS was defined as Δ√transformed CACS divided by the inter-scan period. All computed tomography (CT) scans used to assess CAC were obtained from a > 16-slice multi-detector CT scanner (Philips Brilliance 256 iCT [Philips Healthcare, Cleveland, OH], Philips Brilliance 40-channel multi-detector CT [Philips Healthcare], GE 64-slice Lightspeed [GE Healthcare, Milwaukee, WI], and Siemens 16-slice Sensation [Siemens, Forchheim, Germany]). Standard prospective or retrospective methods were used in all centers. All methods were performed in accordance with the relevant guidelines and regulations. The appropriate institutional review board committees of Severance Cardiovascular Hospital approved the protocol of the present study.

### Statistical analysis

Continuous variables are expressed as the mean ± standard deviation. Categorical variables are expressed as absolute values and percentage. The one-way analysis of variance was used to analyze continuous variables. The χ2 or Fisher’s exact test was used to analyze categorical variables, as appropriate. Univariate linear regression analysis was used to assess the association of clinical variables with the annualized Δ√transformed CACS in each categorical CACS group. Multiple logistic regression models were used to identify the independent predictive value of the AIP for CAC progression beyond that for traditional risk factors according to the baseline CACS status, using the forced entry method. Relative risk was estimated by a log-binomial regression analysis for generalized linear models. All statistical analyses were performed using the Statistical Package for the Social Sciences version 19 (SPSS, Chicago, IL) and SAS version 9.1.3 (SAS Institute Inc., Cary, NC). A *P* value < 0.05 was considered significant in all analyses.

### Ethics approval and consent to participate

The protocol of the present study was approved by the institutional review board of each institution, and written informed consent was obtained from each participant.

## Results

### Baseline characteristics

Clinical characteristics of the 12,326 participants (10,382 men [84.2%]) are presented in Table 1. The mean age of the participants was 51.7 ± 8.5 years. The traditional risk factors of hypertension, diabetes, hyperlipidemia, and current smoking were observed in 33.5%, 13.8%, 28.0%, and 28.5% of participants, respectively. The mean levels of the AIP quartile-stratified groups of I (lowest), II, III, and IV (highest) were 0.007 ± 0.129, 0.274 ± 0.057, 0.469 ± 0.058, and 0.759 ± 0.154, respectively. Systolic and diastolic BP, BMI, the levels of total cholesterol, triglyceride, glucose, and HbA1C, and the prevalence of male sex, hypertension, diabetes, hyperlipidemia, and current smoking were significantly increased with increasing AIP quartile. In contrast, the mean level of HDL-C was significantly decreased with increasing AIP quartile. Clinical characteristics according to CAC progression are described in Supplementary Table 1.

**Table 1:** Clinical characteristics of the study cohort.

	Total (n = 12,326)	Quartiles of AIP				*P*
		I (lowest) (n = 3103) 0.620 − 0.170	II (n = 3064) 0.171 − 0.369	III (n = 3085) 0.370 − 0.575	IV (highest) (n = 3074) 0.576 − 1.738	
Age, years	51.7 ± 8.5	51.6 ± 9.0	52.1 ± 8.6	52.2 ± 8.4	50.9 ± 7.9	< 0.001
Male, n (%)	10,382 (84.2)	2,134 (68.8)	2,573 (84.0)	2,793 (90.5)	2,882 (93.8)	< 0.001
Systolic BP, mmHg	119.6 ± 15.0	117.5 ± 15.1	119.5 ± 15.1	120.1 ± 15.0	121.1 ± 14.6	< 0.001
Diastolic BP, mmHg	75.0 ± 10.5	72.9 ± 10.7	75.0 ± 10.5	75.5 ± 10.4	76.8 ± 10.2	< 0.001
BMI, kg/m^2^	24.6 ± 2.8	23.3 ± 2.7	24.3 ± 2.6	25.0 ± 2.6	25.7 ± 2.6	< 0.001
**Traditional risk factors**						
Hypertension, n (%)	4,016 (33.6)	806 (27.0)	965 (32.6)	1,095 (36.6)	1,150 (38.3)	< 0.001
Diabetes, n (%)	1,703 (13.8)	300 (9.7)	367 (12.0)	502 (16.3)	534 (17.4)	< 0.001
Hyperlipidemia, n (%)	3,455 (28.0)	587 (18.9)	767 (25.0)	926 (30.0)	1,175 (38.2)	< 0.001
Obesity, n (%)	5,178 (42.2)	760 (24.6)	1,153 (37.8)	1,468 (47.8)	1,797 (58.6)	< 0.001
Current smoking, n (%)	3,229 (28.5)	529 (18.6)	669 (23.9)	891 (31.4)	1,140 (40.1)	< 0.001
**Laboratory**						
Total cholesterol, mg/dL	197.5 ± 34.0	191.9 ± 32.1	195.5 ± 33.6	198.0 ± 34.0	204.6 ± 35.1	< 0.001
Triglyceride, mg/dL	141.7 ± 89.4	69.9 ± 19.9	103.2 ± 21.9	143.1 ± 29.7	250.9 ± 108.1	< 0.001
HDL-C, mg/dL	53.3 ± 16.0	68.3 ± 19.3	54.7 ± 10.9	48.5 ± 9.0	41.8 ± 7.7	< 0.001
LDL-C, mg/dL	122.0 ± 31.7	110.2 ± 30.8	123.8 ± 31.0	128.0 ± 30.5	126.1 ± 31.5	< 0.001
Fasting glucose, mg/dL	97.8 ± 20.3	92.9 ± 16.3	96.6 ± 17.8	99.4 ± 20.7	102.3 ± 24.4	< 0.001
HbA1C, %	5.7 ± 0.7	5.5 ± 0.6	5.6 ± 0.7	5.7 ± 0.8	5.8 ± 0.9	< 0.001
AIP	0.377 ± 0.295	0.007 ± 0.129	0.274 ± 0.057	0.469 ± 0.058	0.759 ± 0.154	< 0.001

Values are presented as the mean ± standard deviation or number (%).

*AIP* atherogenic index of plasma*, BMI* body mass index, *BP* blood pressure, *HbA1C* glycated hemoglobin A1C, *HDL-C* high-density lipoprotein cholesterol, *LDL-C* low-density lipoprotein cholesterol.

### Baseline and changes in CAC

**Figure 1:**
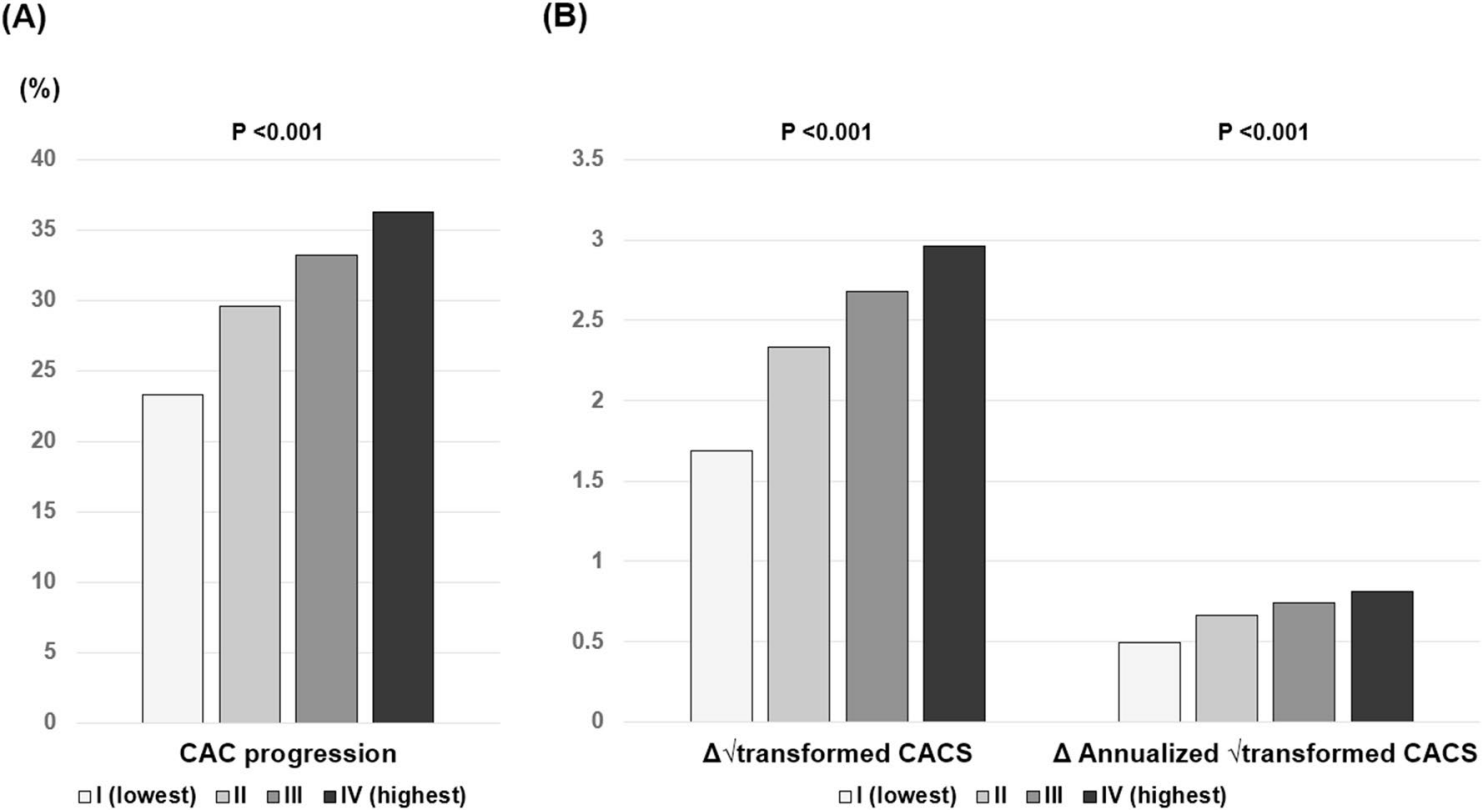
Changes in CAC according to AIP quartile. (**a**) CAC progression; (**b**) Δ√transformed CACS and annualized Δ√transformed CACS. *AIP* atherogenic index of plasma, *CAC* coronary artery calcification, *CACS* coronary artery calcification score.

The overall prevalence of baseline CACSs of 0, 1–100, and > 100 were 56.2%, 33.2%, and 10.6%, respectively. A significant difference in the proportion of categorical CACS among the three groups was observed (Supplementary Fig. 1). During the follow-up period of 3.3 ± 1.8 years, CAC progression was observed in 30.6% of participants. The incidence of CAC progression (group I: 23.3% versus [vs.] group II: 29.6% vs. group III: 33.2% vs. group IV: 36.3%; *P* < 0.001), Δ√transformed CACS (group I: 1.69 ± 3.97 vs. group II: 2.33 ± 4.79 vs. group III: 2.69 ± 4.95 vs. group IV: 2.96 ± 5.06; *P* < 0.001), and annualized Δ√transformed CACS (group I: 0.49 ± 1.48 vs. group II: 0.66 ± 1.64 vs. group III: 0.74 ± 2.18 vs. group IV: 0.81 ± 1.65; *P* < 0.001) were elevated with increasing AIP quartile (Fig. 1).

### AIP and CAC change in each baseline CACS

**Figure 2:**
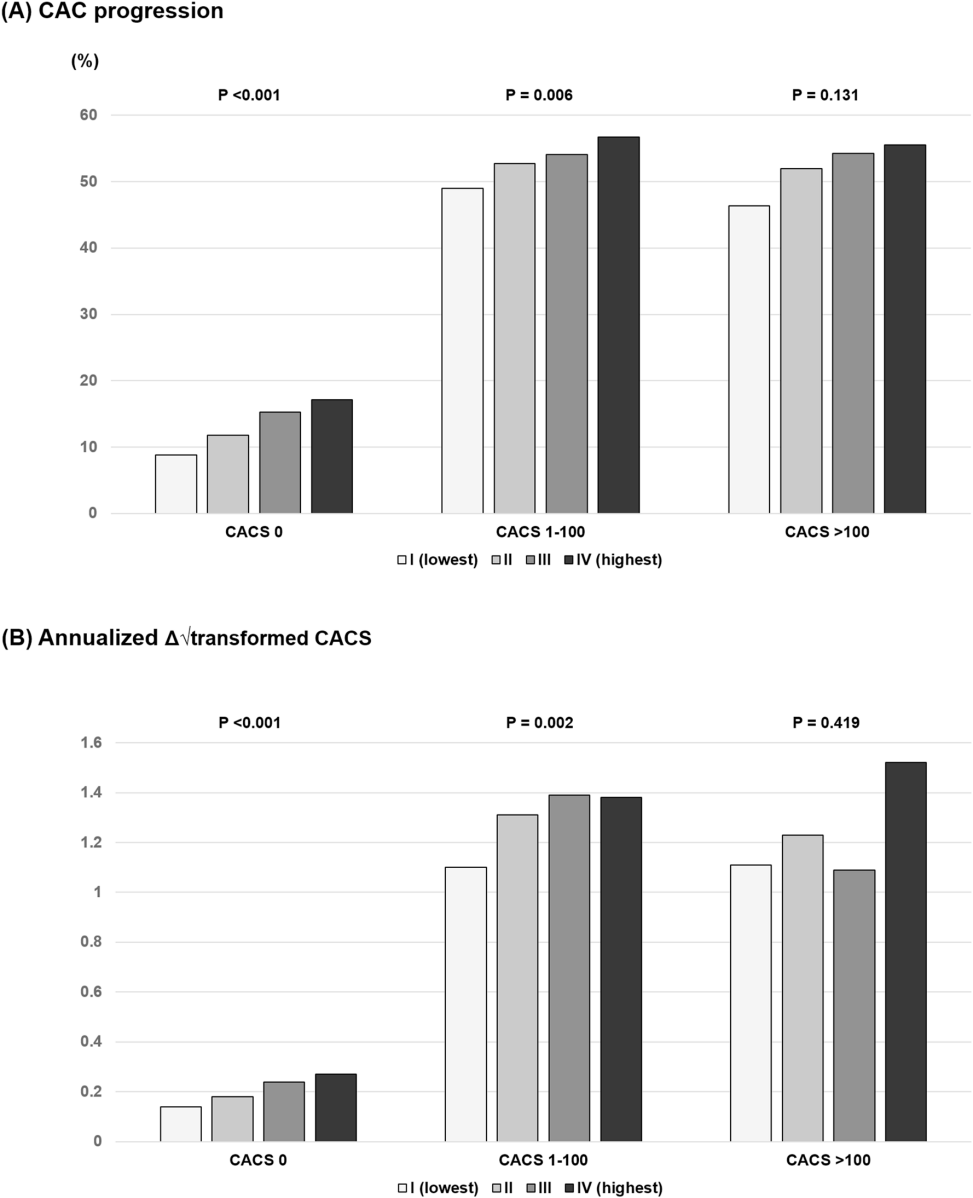
Changes in CAC according to AIP quartile and baseline CACS. (**a**) CAC progression; (**b**) annualized Δ√transformed CACS. *AIP* atherogenic index of plasma, *CAC* coronary artery calcification, *CACS* coronary artery calcium score.

The incidence of CAC progression was elevated with increasing AIP quartile in those with baseline CACSs of 0 (8.8% vs. 11.8% vs. 15.3% vs. 17.1%, *P* < 0.001) and 1–100 (49.0% vs. 52.7% vs. 54.1% vs. 56.7%, *P* = 0.006), but not in those with baseline CACS > 100 (46.4% vs. 52.0% vs. 54.3% vs. 55.5%, *P* = 0.131). The annualized Δ√transformed CACS was significantly different among AIP quartiles in those with baseline CACSs of 0 (0.14 ± 0.52 vs. 0.18 ± 0.59 vs. 0.24 ± 0.71 vs. 0.27 ± 0.72, *P* < 0.001) and 1–100 (1.10 ± 1.63 vs. 1.31 ± 2.00 vs. 1.40 ± 1.97 vs. 1.36 ± 1.71, *P* = 0.002), but not in those with baseline CACS > 100 (1.11 ± 3.53 vs. 1.23 ± 2.80 vs. 1.09 ± 5.01 vs. 1.52 ± 3.08, *P* = 0.419) (Fig. 2).

### AIP and CAC change according to baseline CACS

The AIP (per 0.1-unit increase) was associated with the annualized Δ√transformed CACS in those with baseline CACSs of 0 (β = 0.016; *P* < 0.001) and 1–100 (β = 0.035; *P* < 0.001). However, no significant association between the AIP and annualized Δ√transformed CACS was observed in those with baseline CACS > 100 (β = 0.032; *P* = 0.385) (Table 2). With regards to the association between the AIP and the risk of CAC progression according to the baseline CACS, after adjusting for age, sex, hypertension, diabetes, hyperlipidemia, obesity, and current smoking, multiple logistic regression models showed that the AIP (per 0.1-unit increase) was significantly associated with an increased risk of CAC progression in those with baseline CACSs of 0 and 1–100. However, this association between the AIP and the risk of CAC progression was not observed in those with baseline CACS > 100 (Table 3).

**Table 2:** Clinical variables and annualized Δ√transformed CACS according to baseline CACS.

Variables	CACS 0			CACS 1–100			CACS > 100		
	β	SE	*P*	β	SE	*P*	β	SE	*P*
Age, pre-1 year increase	0.012	0.001	< 0.001	0.016	0.004	< 0.001	0.007	0.012	0.584
Male	0.144	0.019	< 0.001	0.546	0.092	< 0.001	0.724	0.372	0.052
Systolic BP, per-1 mmHg increase	0.003	0.001	< 0.001	− 0.001	0.002	0.692	− 0.005	0.007	0.438
Diastolic BP, per-1 mmHg increase	0.004	0.001	< 0.001	− 0.011	0.003	0.001	− 0.008	0.010	0.440
BMI, per-1 kg/m^2^	0.019	0.003	< 0.001	0.051	0.011	< 0.001	0.058	0.038	0.123
Total cholesterol, per-1 mg/dL increase	0.001	0.001	< 0.001	− 0.002	0.001	0.020	0.001	0.003	0.953
Triglyceride, per-1 mg/dL increase	0.001	0.001	< 0.001	0.001	0.001	< 0.001	0.001	0.001	0.268
HDL-C, per-1 mg/dL increase	− 0.001	0.001	0.053	0.001	0.002	0.583	0.001	0.007	0.940
LDL-C, per-1 mg/dL increase	0.001	0.001	< 0.001	0.001	0.001	0.359	0.001	0.003	0.774
AIP, per-0.1 unit increase	0.016	0.003	< 0.001	0.035	0.010	< 0.001	0.032	0.037	0.385
Fasting glucose, per-1 mg/dL increase	0.004	0.001	< 0.001	0.007	0.001	< 0.001	0.001	0.004	0.941
Current smoking	0.159	0.045	< 0.001	1.028	0.177	< 0.001	2.107	0.514	< 0.001

*AIP* atherogenic index of plasma*, BMI* body mass index, *BP* blood pressure, *CACS* coronary artery calcium score, *HDL-C* high-density lipoprotein cholesterol, *LDL-C* low-density lipoprotein cholesterol.

**Table 3:** Impact of the AIP (per 0.1-unit increase) on CAC progression, beyond traditional risk factors, according to baseline categorical CACS.

	OR (95% CI)	*P*	RR (95% CI)	*P*
**CACS 0**				
Model 1	1.10 (1.08–1.13)	< 0.001	1.09 (1.07–1.11)	< 0.001
Model 2	1.05 (1.02–1.08)	0.001	1.04 (1.02–1.06)	0.001
**CACS 1–100**				
Model 1	1.04 (1.02–1.07)	< 0.001	1.02 (1.01–1.03)	< 0.001
Model 2	1.03 (1.01–1.05)	0.022	1.01 (1.01–1.02)	0.020
**CACS > 100**				
Model 1	1.05 (1.01–1.09)	0.019	1.02 (1.01–1.04)	0.018
Model 2	1.00 (0.96–1.05)	0.940	1.00 (0.98–1.02)	0.928

Models: 1 = unadjusted; 2 = adjusted for age, sex, and the traditional risk factors of hypertension, diabetes, hyperlipidemia, obesity, and current smoking.

*AIP* atherogenic index of plasma, *BMI* body mass index, *CAC* coronary artery calcification, *CACS* coronary artery calcium score, *CI* confidence interval, *OR* odds ratio, *RR* relative risk.

## Discussion

The present study demonstrated that, during the mean 3.3-year follow-up period, the AIP level was significantly related to the annualized Δ√transformed CACS in participants with baseline CACS ≤ 100 in 12,326 participants from the asymptomatic Korean population. After adjusting for traditional risk factors, the AIP was significantly associated with an increased risk of CAC progression in these participants.

Budoff et al. previously reported that CAC progression had additive value in predicting all-cause mortality, beyond that for the baseline score and CV risk factors, in 4,609 asymptomatic participants who underwent repeat screening during a mean 3.1-year period^4^. In contrast, the CCLS (Cooper Center Longitudinal Study) trial found only a modest association between CAC progression and CV outcomes, although additional prognostic information was not provided in comparison with that for the latest single CAC value, during the mean 3.5-year period^16^. Lehmann et al. recently reported that CAC progression may only add prognostic benefit for participants without CV disease, based on the HNR study data (n = 3,281), during a mean 5.1-year follow-up^5^. This previous study emphasized the significance of the most recent CAC value and risk factor assessment. Additional CAC examination is not required in cases of heavy CAC at baseline, which implies the presence of a high CV risk. Considering that atherosclerosis-related adverse events commonly occur, even in populations with low CV risk burden^17–19^, these results strongly suggest that early detection of the presence and progression of subclinical atherosclerosis is an important issue in clinical practice.

Previous studies revealed that the AIP is significantly related to diverse metabolic disorders, such as fatty liver disease, hypertension, and diabetes^20,21^. In addition, a number of cross-sectional studies demonstrated that the AIP had predictive value for coronary artery disease in various clinical conditions^22–24^. However, earlier studies did not address the relationship between the AIP and CAC progression, especially in terms of the baseline CACS. In the present study, we considered a CACS > 100 as indicating heavy CAC status based on the following facts: (1) Nasir et al.^25^ reported that the frequency of CACS > 100 in the Asian population is significantly lower than that in western populations; and (2) the overall proportion of baseline CACS > 100 was 10.6% in our participants. The presence of CAC at baseline and CAC progression during follow-up were more frequently observed with increasing AIP quartile. However, the AIP was associated with the risk of CAC progression, over that for traditional CV risk factors, in participants without heavy CAC at baseline. This finding suggests that the AIP is a useful marker for the stratification of CV risk, in addition to the baseline CAC status, in conditions with low to intermediate CV risk.

Because the present study only evaluated the change in CACS in the asymptomatic adult population, data on changes in the regional distribution or pattern of CAC were unavailable. In addition, the effect of medications such as statins, which could influence the changes in plaque sub-types, was not considered^26^. However, a recent CONFIRM (COronary CT Angiography EvaluatioN For Clinical Outcomes: An InteRnational Multicenter) sub-study found that further prognostic benefit was not offered by coronary computed tomographic angiography (CCTA) findings when added to traditional risk factors and CACS in 1,226 asymptomatic participants, during a mean 5.9 ± 1.2 years of follow-up^27^. This finding suggests that CCTA may have a limited role in future CV risk stratification in the asymptomatic population, until further proven.

We should acknowledge some limitations of the present study. First, this study included participants from the KOICA registry who underwent at least two CAC scans with available data on the AIP and diabetic status. Thus, a potential selection bias might be present. Second, we only identified the association between the baseline AIP level and CAC progression, focusing on the baseline CACS; consecutive changes in AIP during the follow-up period were not confirmed. However, the mean change in the AIP between the initial and follow-up CAC scan was very small: − 0.02 ± 0.23. Third, the use of medications for hypertension, diabetes, and hyperlipidemia was not controlled during the follow-up period because this study had an observational design. Fourth, although we evaluated the predictive value of the AIP for CAC progression after adjusting for sex, the predominance of male sex in this cohort registry could have impacted the results. Thus, it might be hard to apply the results of present study to the female population. Fifth, although this study was performed in an asymptomatic and relatively healthy population, there was a paucity of data on environmental risk factors, such as physical activity, exercise, and diet, which could influence CAC progression. Sixth, different CT scanners were used among the participating centers because the KOICA registry was a retrospective, observational, and multicenter registry. However, all participants were examined using the identical CT scanner at both the initial and follow-up image acquisitions. Finally, the generalizability of our results may be limited considering that all participants were Korean. Despite these limitations, we firstly identified the predictive value of the AIP for CAC progression according to the baseline CACS in a large sample of the asymptomatic adult population.

## Conclusion

In summary, the present study demonstrates that the AIP is independently associated with CAC progression, especially in the absence of heavy CAC at baseline. This result suggests that the AIP has the potential to predict early coronary atherosclerotic changes and to stratify CV risk in asymptomatic adults with low to intermediate CV risk. Further large-scale prospective studies are necessary to confirm the significance of the AIP in primary prevention.

## Supplementary information

Supplementary Information 1.

## Data Availability

The datasets used and analyzed during the current study are available from the corresponding author on reasonable request.
