# Supplementary material for: Atherogenic index of plasma and coronary artery calcification progression beyond traditional risk factors according to baseline coronary artery calcium score
Source: Sci Rep. 2020 Dec 7;10:21324. doi: 10.1038/s41598-020-78350-x (PMC7721801; doi:10.1038/s41598-020-78350-x)
Supplement: Supplementary file 1 — Supplementary Information 1. [file 41598_2020_78350_MOESM1_ESM.pdf]

Atherogenic index of plasma and coronary artery calcification progression beyond traditional risk factors according to baseline coronary artery calcium score

Ki-Bum Won[1,2], Donghee Han[2,3], Ji Hyun Lee[4], Su-Yeon Choi[5], Eun Ju Chun[6], Sung Hak Park[7], Hae-Won Han[8], Jidong Sung[9], Hae Ok Jung[10], Hyuk-Jae Chang[2]

Affiliations:

[1]Division of Cardiology, Ulsan University Hospital, University of Ulsan College of Medicine, Ulsan, South Korea

[2]Division of Cardiology, Yonsei Cardiovascular Center, Yonsei University Health System, South Korea

[3]Division of Cardiology, New York-Presbyterian Hospital and Weill Cornell Medical College, New York, NY, USA

[4]Division of Cardiology, Myongji Hospital, Ilsan, South Korea

[5]Division of Cardiology, Healthcare System Gangnam Center, Seoul National University Hospital, Seoul, Korea

[6]Division of Radiology, Seoul National University Bundang Hospital, Seongnam, Korea

[7]Division of Radiology, Gangnam Heartscan Clinic, Seoul, Korea

[8]Department of Internal Medicine, Gangnam Heartscan Clinic, Seoul, Korea

[9]Division of Cardiology, Heart Stroke & Vascular Institute, Samsung Medical Center, Seoul, Korea

[10]Division of Cardiology, Seoul St. Mary's Hospital, College of Medicine, The Catholic University of Korea, Seoul, Korea

E-mail:

Ki-Bum Won, E-mail: kbwon99@naver.com

Donghee Han, E-mail: ibanezera@gmail.com

Ji Hyun Lee, E-mail: jihyun.cardio@gmail.com

Su-Yeon Choi, E-mail: sychoi9@gmail.com

Eun Ju Chun, E-mail: drejchun@hanmail.net

Sung Hak Park, E-mail: shparkmdmax@gmail.com

Hae-Won Han, E-mail: drhanhae@gmail.com

Jidong Sung, E-mail: jidong.sung@gmail.com

Hae Ok Jung, E-mail: hojheart@catholic.ac.kr

Hyuk-Jae Chang, E-mail: hjchang@yuhs.ac

Running title: AIP and CAC progression

Conflicts of Interest: The authors have no conflicts of interest to declare.

Address for correspondence:

Hyuk-Jae Chang, MD, PhD,

Division of Cardiology, Severance Cardiovascular Hospital,

Yonsei-Cedars-Sinai Integrative Cardiovascular Imaging Research Center,

Yonsei University College of Medicine,

Yonsei University Health System,

50-1 Yonsei-ro, Seodaemun-gu, Seoul 03722, South Korea

Tel.: +82(0)2 22288460; Fax: +82(0)2 3932041

E-mail: hjchang@yuhs.ac

FIGURE LEGENDS

Figure 1. Changes in CAC according to AIP quartile

(a) CAC progression; (b) Δ√transformed CACS and annualized Δ√transformed CACS

AIP atherogenic index of plasma, *CAC* coronary artery calcification, *CACS* coronary artery calcification score

Figure 2. Changes in CAC according to AIP quartile and baseline CACS

(a) CAC progression; (b) annualized Δ√transformed CACS

AIP atherogenic index of plasma, *CAC* coronary artery calcification, *CACS* coronary artery calcium score

SUPPLEMENTARY APPENDIX

Supplementary Figure 1. Baseline CACS according to AIP quartiles

AIP atherogenic index of plasma, *CACS* coronary artery calcium score

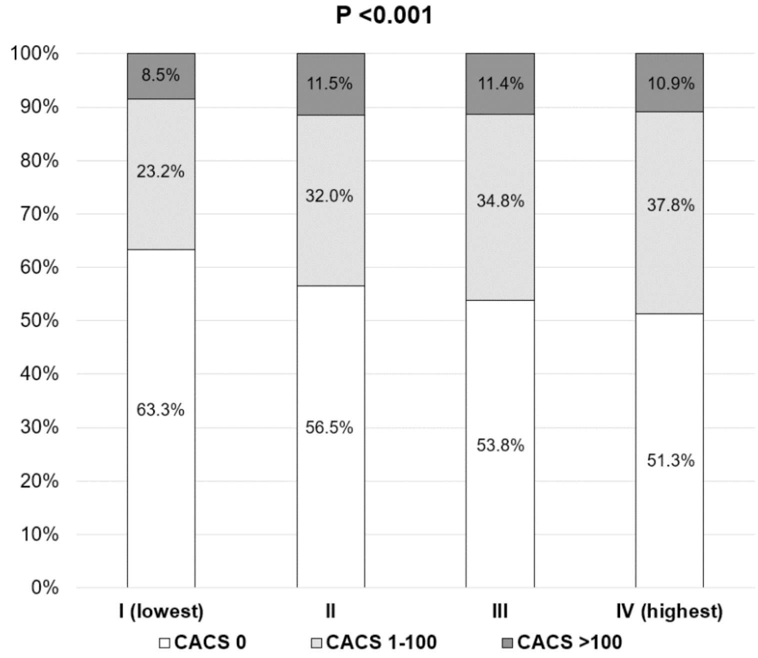

Supplementary Table 1. Clinical characteristics according to CAC progression

	No CAC progression (n = 8,557)	CAC progression (n = 3,769)	P
Age, years	50.2 ± 8.2	55.0 ± 8.1	<0.001
Male, n (%)	6,924 (80.9)	3,458 (91.7)	<0.001
Systolic BP, mmHg	118.9 ± 14.8	121.1 ± 15.4	<0.001
Diastolic BP, mmHg	74.8 ± 10.6	75.7 ± 10.4	<0.001
BMI, kg/m^2	24.3 ±2.8	25.0 ± 2.7	<0.001
Traditional risk factors			
Hypertension, n (%)	2,334 (28.3)	1,682 (45.5)	<0.001
Diabetes, n (%)	904 (10.6)	799 (21.2)	<0.001
Hyperlipidemia, n (%)	2,100 (24.5)	1,355 (36.0)	<0.001
Obesity, n (%)	3,319 (39.0)	1,859 (49.4)	<0.001
Current smoking, n (%)	2,195 (28.0)	1,034 (29.7)	0.054
Laboratory			
Total cholesterol, mg/dL	197.1 ± 33.7	198.3 ± 34.9	<0.001
Triglyceride, mg/dL	136.0 ± 87.2	154.6 ± 93.0	<0.001
HDL-C, mg/dL	53.8 ± 16.4	52.4 ± 14.9	<0.001
LDL-C, mg/dL	120.5 ± 31.4	125.2 ± 32.2	<0.001

Fasting glucose, mg/dL	96.1 ± 18.6	101.6 ± 23.3	<0.001
HbA1C, %	5.6 ± 0.7	5.8 ± 0.8	<0.001
AIP	0.355 ± 0.298	0.425 ± 0.284	<0.001

Values are presented as the mean ± standard deviation or number (%).

AIP atherogenic index of plasma, *BMI* body mass index, *BP* blood pressure, *HbA1C* glycated hemoglobin A1C, *HDL-C* high-density lipoprotein cholesterol, *LDL-C* low-density lipoprotein cholesterol
